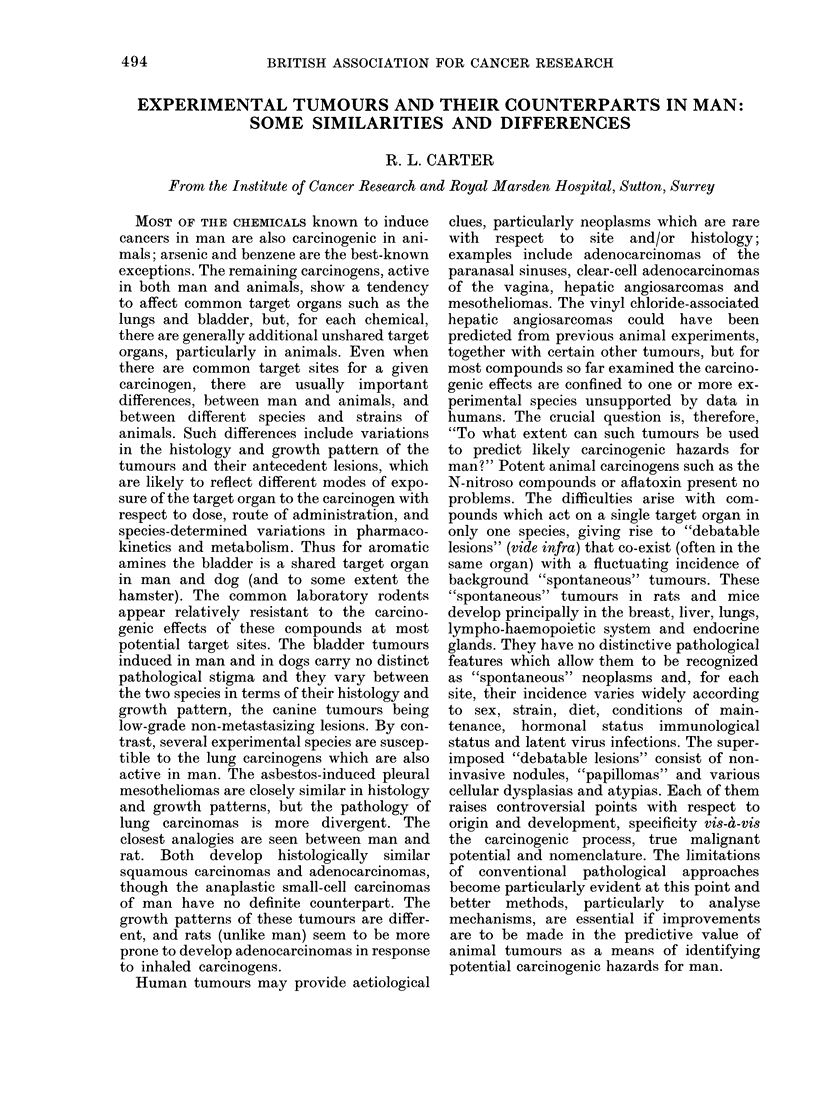# Experimental tumours and their counterparts in man: some similarities and differences.

**DOI:** 10.1038/bjc.1980.77

**Published:** 1980-03

**Authors:** R. L. Carter


### EXPERIMENTAL TUMOURS AND THEIR COUNTERPARTS IN MAN: SOME SIMILARITIES AND DIFFERENCES

R. L. CARTER

*From the Institute of Cancer Research and Royal Marsden Hospital, Sutton, Surrey*

MOST OF THE CHEMICALS known to induce cancers in man are also carcinogenic in animals; arsenic and benzene are the best-known exceptions. The remaining carcinogens, active in both man and animals, show a tendency to affect common target organs such as the lungs and bladder, but, for each chemical, there are generally additional unshared target organs, particularly in animals. Even when there are common target sites for a given carcinogen, there are usually important differences, between man and animals, and between different species and strains of animals. Such differences include variations in the histology and growth pattern of the tumours and their antecedent lesions, which are likely to reflect different modes of exposure of the target organ to the carcinogen with respect to dose, route of administration, and species-determined variations in pharmacokinetics and metabolism. Thus for aromatic amines the bladder is a shared target organ in man and dog (and to some extent the hamster). The common laboratory rodents appear relatively resistant to the carcinogenic effects of these compounds at most potential target sites. The bladder tumours induced in man and in dogs carry no distinct pathological stigma and they vary between the two species in terms of their histology and growth pattern, the canine tumours being low-grade non-metastasizing lesions. By contrast, several experimental species are susceptible to the lung carcinogens which are also active in man. The asbestos-induced pleural mesotheliomas are closely similar in histology and growth patterns, but the pathology of lung carcinomas is more divergent. The closest analogies are seen between man and rat. Both develop histologically similar squamous carcinomas and adenocarcinomas, though the anaplastic small-cell carcinomas of man have no definite counterpart. The growth patterns of these tumours are different, and rats (unlike man) seem to be more prone to develop adenocarcinomas in response to inhaled carcinogens.

Human tumours may provide aetiological clues, particularly neoplasms which are rare with respect to site and/or histology; examples include adenocarcinomas of the paranasal sinuses, clear-cell adenocarcinomas of the vagina, hepatic angiosarcomas and mesotheliomas. The vinyl chloride-associated hepatic angiosarcomas could have been predicted from previous animal experiments, together with certain other tumours, but for most compounds so far examined the carcinogenic effects are confined to one or more experimental species unsupported by data in humans. The crucial question is, therefore, "To what extent can such tumours be used to predict likely carcinogenic hazards for man?" Potent animal carcinogens such as the N-nitroso compounds or aflatoxin present no problems. The difficulties arise with compounds which act on a single target organ in only one species, giving rise to "debatable lesions" (*vide infra*) that co-exist (often in the same organ) with a fluctuating incidence of background "spontaneous" tumours. These "spontaneous" tumours in rats and mice develop principally in the breast, liver, lungs, lympho-haemopoietic system and endocrine glands. They have no distinctive pathological features which allow them to be recognized as "spontaneous" neoplasms and, for each site, their incidence varies widely according to sex, strain, diet, conditions of maintenance, hormonal status immunological status and latent virus infections. The superimposed "debatable lesions" consist of non-invasive nodules, "papillomas" and various cellular dysplasias and atypias. Each of them raises controversial points with respect to origin and development, specificity *vis-à-vis* the carcinogenic process, true malignant potential and nomenclature. The limitations of conventional pathological approaches become particularly evident at this point and better methods, particularly to analyse mechanisms, are essential if improvements are to be made in the predictive value of animal tumours as a means of identifying potential carcinogenic hazards for man.